# Exploring the Experiences of Transgender and Gender Diverse Adults in Accessing a Trans Knowledgeable Primary Care Physician

**DOI:** 10.3390/ijerph182413057

**Published:** 2021-12-10

**Authors:** Shanna K. Kattari, Jarrod Call, Brendon T. Holloway, Leonardo Kattari, Kristie L. Seelman

**Affiliations:** 1 Department of Women’s and Gender Studies, School of Social Work, University of Michigan, Ann Arbor, MI 48109, USA; 2 Graduate School of Social Work, University of Denver, Denver, CO 80208, USA; jarrod.call@du.edu (J.C.); Brendon.Holloway@du.edu (B.T.H.); 3 School of Social Work, Michigan State University, East Lansing, MI 48824, USA; kattaril@msu.edu; 4 School of Social Work, Georgia State University, Atlanta, GA 30302, USA; kseelman@gsu.edu

**Keywords:** health, transgender, gender diverse, primary care, healthcare, gender identity, United States

## Abstract

Transgender and gender diverse individuals face a variety of barriers when attempting to access healthcare, from discrimination to lack of access to lack of knowledgeable providers. Using data from the 2015 United States Trans Survey (*N* = 27,715), this study looks at the differences within the TGD population regarding having seen a doctor in the past year, having a primary care provider, and having a primary care provider who is knowledgeable about trans health. Logistic regressions indicate that even within an all transgender and gender diverse sample, a variety of identities and experiences are related to increased or decreased likelihood of each of these outcomes, with significant differences across gender, race/ethnicity, age, sexual orientation, disability status, educational attainment, annual income, disability status, religiosity, military status, overall health, housing status, and insurance coverage. Not only should there be an effort to support transgender and gender diverse individuals in accessing care, but there is a clearly indicated need for additional education for healthcare providers, especially those doing primary care, on how to offer knowledgeable, affirming, and intersectional care to their patients.

## 1. Introduction

Research has shown that transgender and gender diverse (TGD) individuals experience a variety of challenges when it comes to accessing healthcare. Some of these issues are due to transphobia, and many TGD patients have experienced discrimination, harassment, and even physical harm at the hands of healthcare providers [1,2,3]. Others may delay care for fear of these things, due to lack of insurance and/or funds, not having a trans knowledgeable provider nearby, or a plethora of other reasons. A trans knowledgeable provider is someone who is not only respectful to their TGD patients through using their correct name and pronouns, but one that also has knowledge of the unique health needs facing TGD individuals, such as hormone prescriptions (and interactions) and gender-affirming surgery options and referrals, and also recognizes that TGD individuals will experience similar concerns to their cisgender patients, such as having the flu, or needing a broken arm to be healed. However, the TGD population is not a monolithic group, and so even under the TGD umbrella, there are differences in who is able to go to the doctor, who has a primary care provider (PCP), and whose PCPs are knowledgeable about issues related to TGD health and healthcare. This study explores within group differences across these three outcomes.

We use the phrase TGD to encompass those whose gender differs from hegemonic White/European social expectations related to the sex that they were assigned at birth. This umbrella term includes individuals who may transition from one gender to another—such as transgender women and transgender men—as well as those who do not identify within a gender binary, including those who identify as genderqueer, two-spirit (an identity specific to Indigenous individuals), nonbinary, agender, etc.

***Access to Care***. Transgender and gender diverse people (TGD) experience negative health outcomes because of systemic oppression [1], and are often required to navigate a healthcare system that can be openly hostile toward TGD individuals [2]. A primary reason for these negative outcomes is that TGD individuals often do not have or have difficulty accessing healthcare. The extant literature shows that TGD adults are less likely to access primary and specialty healthcare services than their cisgender (non-transgender) peers [3]. Among TGD young adults, 35% received a routine check-up in the past year compared to 49%–58% of cisgender young adults [4,5]. Additionally, a study based in Philadelphia found that more than 50% of TGD participants experienced challenges when accessing one or more healthcare services in the past year [6]. 

A common barrier for TGD people when attempting to access care is inadequately trained medical providers [7]. This occurs across health specialties, including primary care, surgical care, obstetrics and gynecology, and endocrinology [8,9]. As a result, TGD people are often burdened with teaching their medical providers about their health, leading many to feel alienated by the medical system due to their healthcare needs not being met [10]. Findings from the Virginia Transgender Initiative Health study, a study that surveyed 350 TGD individuals, showed an alarming lack of TGD-friendly and competent providers [11], and results from the National Transgender Discrimination Survey (*N* = 6450) indicated that 50% of TGD respondents had to educate their medical providers about basic transgender health information [12]. Due to the lack of adequately trained medical providers, TGD people are unable to find healthcare that meets their needs [13,14].

Insurance issues are often another barrier for TGD people attempting to access healthcare services. Not having health insurance is tied to higher rates of healthcare harassment and discrimination [15]. In the United States, 14% of TGD people do not have health insurance compared to 11% of cisgender people [16]. This is amplified for people of color, as one study found that 25.7% of TGD people of color did not have health insurance compared to 17% of white TGD people [17]. Even when TGD people do have health insurance that supposedly covers gender-affirming care, insurance companies often set payout limits below the cost of needed gender-affirmation procedures or refuse to pay for these procedures, resulting in covered care being unattainable for many [18]. Additionally, most people in the U.S. receive health insurance through their employers [19], which creates additional barriers for TGD individuals since they are more likely to experience discrimination in the workplace and unemployment than cisgender individuals [16]. 

***Sexual orientation***. Generally, among TGD populations, individuals describe their sexual attractions, identities, and behaviors more expansively than among cisgender populations. For example, in the TransPop Survey, which used a national probability sample of transgender adults and compared them to a random sample of cisgender adults, among the most common sexual orientations among the transgender groups were bisexual, queer, and pansexual, as well as heterosexual [20]. In contrast, cisgender adults are most likely to say that they are heterosexual, bisexual, gay, or lesbian [21,22]. Past research suggests that TGD individuals who have a sexual orientation other than heterosexual (or, in some cases, asexual) tend to be more likely to experience mistreatment in healthcare. In an analysis of the experiences of transgender men within the United States Transgender Survey (USTS) [23], queer transgender men were more likely to have to teach a doctor about transgender-related issues, be asked invasive questions, and experience any mistreatment in healthcare compared to most other orientation groups. Meanwhile, asexual and heterosexual transgender men were less likely to be asked invasive questions or have a doctor use harsh or abusive language compared to bi/pansexuals and those with an orientation not listed on the survey [23]. In another paper using USTS data [24], TGD individuals who were asexual had the lowest risks for mistreatment in healthcare, followed by bi/pansexual and heterosexual individuals; those who were lesbian, gay, same-gender-loving, queer, or an orientation not listed had the greatest risks of poor treatment by healthcare providers. This evidence suggests that LGBQ + TGD patients may have less access to culturally responsive, knowledgeable healthcare providers and/or experience a greater risk of discrimination in healthcare than heterosexual TGD counterparts.

***Race***. In terms of race, people of color in general are more likely to experience forms of discrimination in healthcare settings compared to their white counterparts [25]. When examining the intersections of gender and race in terms of healthcare access, TGD individuals of color are more likely to experience refusal of care and discrimination in healthcare settings than their white TGD peers [15]. This discrimination occurs in emergency rooms and with doctors, hospitals, and EMTs [26]. Additionally, TGD feminine people of color experience much higher rates of refusal of care from providers than TGD white individuals and TGD masculine people of color [27]. Moreover, TGD men and women who are multiracial and TGD men who are Native American are more likely to delay medical care due to discrimination [28]. 

***Disability status***. Shifting to disability status, according to the 2010 U.S. Census Bureau [29], 21.3% of people over 15 years old had a disability or impairment. Although nearly one quarter of the U.S. population has a disability or impairment, disabled people are significantly more likely to experience victimization in many settings [30], including high rates of discrimination in healthcare [31,32]. When looking at the intersections of gender identity and disability, TGD disabled adults experience higher rates of discrimination in mental healthcare settings. In the same study, TGD disabled adults with multiple disabilities and impairments were significantly more likely to have experienced discrimination than their non-disabled peers [33]. 

***Age***. Although there is limited healthcare research regarding the intersection of TGD individuals and age, similar patterns of adverse health outcomes that impact young and middle-aged TGD adults persist into older adulthood [34,35]. The existing literature shows that LGBT older adults report more chronic health conditions and poorer health-related quality of life than heterosexual, cisgender older adults [36]. TGD older adults have poorer health in terms of physical health, mental health, disability, and perceived stress compared to non-TGD older adults [37]. In addition to poor health outcomes, 40% of TGD older adults report receiving poor quality of care or being denied healthcare because of their sexual orientation and/or gender identity [38]. 

***Education level***. In looking at education level, there is not a lot of research examining the intersections of education level, gender identity, and healthcare experiences. According to a 2015 study with TGD men, having a graduate degree was associated with increased reports of discrimination in healthcare settings [39]. This study concluded that stigma and power play a role in healthcare interactions, and that this finding may explain the relationship between reporting discrimination and higher education levels. Jaffee et al. [28] also found that education level influences the choice to delay healthcare due to fear of discrimination. Additionally, those with less than a high school education have a greater odds of experiencing unwanted sexual contact, being verbally harassed, and experiencing victimization in healthcare settings than those with a bachelor’s degree [40]. 

***Insecure housing/homelessness***. Much extant literature on housing insecurity and homelessness among the TGD population centers around the experiences of TGD youth, as found in multiple systematic reviews [41,42]. Limited research on housing insecurity among TGD adults provides exploratory associations between health and wellness and experiencing homelessness. Multiple studies have explored the link between behavioral health outcomes, such as higher likelihood of depression, substance use, and suicidality among TGD individuals experiencing housing insecurity [41,42,43]. One qualitative study found that housing insecurity among TGD individuals is associated with having multiple marginalized identities, experiences of discrimination, and the challenges of accessing healthcare when houseless [43]. 

***Religiosity***. Limited research exists that explores religiosity among TGD individuals. One study exploring unprotected sexual behaviors among transgender women found that those with higher levels of religiosity were more likely to engage in unprotected sex [44]. Another study explored the impact of religious loss or rejection from a grief framework, noting that many TGD individuals feel that they must choose between their faith and their gender. This forced decision to give up religion may cause psychological distress [45]. Much more research is necessary to understand the associations between health and religiosity among TGD individuals. 

***Military status***. Members of the military, and especially veterans of the U.S. military, have had many challenges experiencing discrimination through military healthcare and at the VA [46,47]. There is a paucity of research in this area, and more research is needed to explore how this impacts the health of active service members and military veterans.

### Research Question

TGD individuals have many different experiences when trying to access healthcare, depending on their other identities and life experiences, yet little is known about how sociodemographic factors and these experiences may be connected to being able to access healthcare providers. Based on this gap, this study addresses three questions, while controlling for sociodemographic factors (e.g., gender, sexual orientation, race, age, class, and disability) and other experiences (e.g., educational level, religiosity, whether they have secure housing, military status): (1) how many TGD individuals see a physician on a yearly basis; (2) how many TGD individuals have a regular primary care provider (PCP); and (3) how many of these individuals have a PCP who they would consider to be knowledgeable about transgender health? 

## 2. Materials and Methods

### 2.1. Design 

The present study conducted a secondary data analysis on the 2015 United States Transgender Survey (USTS), which surveyed 27,715 TGD adults across the United States. The survey was available online in both English and Spanish, and participants were recruited via convenience sampling online and through organizational partners. Respondents provided information about a variety of topics, including demographics, healthcare access history, and how knowledgeable healthcare professionals were of TGD health and health needs. An in-depth description of survey methods can be found in the report by James et al. [16]. Data were provided to the first author, analysis occurred in 2021, and IRB exemption was ascertained by the first author. 

### 2.2. Measures 

*Dependent Variables*. The first dependent variable in this study was the dichotomous yes/no question: “In the past year, have you seen a doctor or healthcare provider?”. The next dependent variable determined if participants had a primary care physician (PCP). This variable was created through two questions. The first, “How much does your routine healthcare provider (who you see for physicals, flu, diabetes, etc.) know about healthcare for trans people”, had response options of “I don’t have a routine healthcare provider”, “They know almost everything about trans healthcare”, “They know most things”, “They know some things”, “They know almost nothing”, and “I am not sure”. Due to survey skip logic, this question was not asked of participants who indicated not currently having a trans-related doctor or provider. To represent participants who did have a provider for trans-related healthcare, the following question was also used to construct the second dependent variable: “Do you also go to your trans-related healthcare provider for your routine healthcare, like physicals, flu, diabetes, etc.?”. Response options included “Yes, I see my trans healthcare provider for my routine healthcare”, “No, I see a different doctor or healthcare provider for my routine healthcare”, and “No, I do not get any routine healthcare”. 

The newly created PCP variable was coded as a dichotomous yes/no variable. The third dependent variable assessed participant perception of how knowledgeable their PCP was about trans care. This variable was created by combining the question “How much does your routine healthcare provider (who you see for physicals, flu, diabetes, etc.) know about healthcare for trans people?” and “Thinking about the doctor or provider you go to for your trans-related healthcare (such as hormone treatment), how much do they know about providing healthcare for trans people?”. The values for the original questions were “I don’t have a routine healthcare provider” or “I don’t have a trans-related doctor or healthcare provider right now”, “They know almost everything about trans healthcare”, “They know most things”, “They know some things”, “They know almost nothing”, and “I am not sure”. 

*Independent Variables*. Independent variables included gender, sexual orientation, age, education, insurance coverage, income, ability status, military history, race/ethnicity, overall health, housing, and religiosity. To avoid misrepresenting identities, the labels used in the original questionnaire for gender were kept the same. This question asked, “If you had to choose only one of the following terms, which best describes your current gender identity?”. Response options included cross-dresser, woman, man, trans woman, trans man, and non-binary/genderqueer. Age was collected as a continuous variable and remained the same for this analysis. Ability was measured through a single dichotomous “yes” or “no” question: “Do you identify as a person with a disability?”. Religiosity was measured by the question, “What is your current religious or spiritual identity?”. Participants who reported being atheist, agnostic, or having no religious or spiritual affiliation were coded as not religious/spiritual, while those who marked any other religious identity were coded as religious/spiritual.

Sexual orientation was measured through the question, “What best describes your current sexual orientation?”. For analytic purposes, asexual and demisexual were combined into one larger asexual/demisexual value, and same-gender loving was included with additional orientations.

Race and ethnicity were assessed through the question, “Although the choices listed below may not represent your full identity or use the language you prefer, for this survey please select the choice that most accurately describes your racial/ethnic identity. (Please choose only one answer)”. Alaskan Native, Middle Eastern/North African, and Native Hawaiian/Pacific Islander were combined with the additional racial/ethnic identity category due to small sample sizes.

Military history was assessed through the question, “Have you ever served on active duty in the U.S. Armed Forces, Reserves, or National Guard?”. This variable was recoded into a dichotomous “no” for those that selected “never served in the military” or “yes” for all other responses.

Education was measured through the question, “What is the highest level of school or degree you have completed?”. These values were recoded into the final values of “Less than high school or GED”, “High school or GED”, “Associate degree (vocational or academic)”, “Some college”, “Bachelor’s”, “Some grad school”, “Master’s”, and “Doctoral or professional degree”.

Housing was measured through the question, “What are your current living arrangements?”. Response options were recoded into three values: “Personal housing”, “Congregate housing”, and “Insecure housing”. 

Household income was determined through the question “How much was your total combined household income (before taxes) in 2014?”. Response options included “No income”, “$1 to $9999”, “10,000 to 24,999”, “25,500 to 49,999”, “50,000 to 99,999”, “100,000 or more.” 

Health insurance was measured by the question, “What type of health insurance or health coverage plan do you have? (Mark all that apply)”. This variable was recoded into public insurance, private insurance, and military insurance. Participants who marked multiple types of insurance were coded as having multiple insurances. Private insurance was used as the reference category. Health status was assessed through the question “Would you say that in general your health is…”. Response options were “Excellent”, “Very good”, “Good”, “Fair”, and “Poor.” 

### 2.3. Data Analysis

Basic descriptive statistics were used to summarize participant attributes. Weighted logistic regression analyses were conducted on the full sample to identify correlates of having visited a physician in the past year, as well as whether participants had a PCP. Ordered logistic regression was then used to examine factors related to PCP TGD health knowledge. Participants who indicated that they did not have a PCP or were uncertain of how knowledgeable their PCP was about TGD health were excluded from this analysis. 

Of the 27,715 participants in the sample, 2934 (10.59%) had missing data on one or more variables. The percentage of cases missing ranged from 0% for gender to 8.7% for household income. Multiple imputation was used to address missingness and prevent loss of power associated with listwise deletion [48]. The *mi chained* command was used in Stata 16 to generate 15 imputed datasets, which is in line with guidelines recommending that the number of imputed datasets be greater than the percentage of incomplete cases [49].

The USTS data used weighting to correct for an overrepresentation of participants who were 18 years old and white to better align with TGD population characteristics demonstrated in other studies [16]. Recommended sampling weights were used in regression analysis. All statistical analyses were conducted using Stata 16 [50].

## 3. Results

### 3.1. Demographics

Participants’ demographics are detailed in Table 1. Nonbinary/genderqueer (34.2%) was the most frequently reported gender, followed by transgender men (21.1%), transgender women (20.3%), women (13.5%), men (8.3%), and cross-dressers (2.6%). A variety of sexual orientations were reported, including queer (20.6%), pansexual (18.2%), bisexual (14.9%), straight (12.1%), asexual/demisexual (11.8%), lesbian (11.0%), and gay (4.8%). The remaining 6.6% of the sample identified with additional orientations not included in the survey. Most of the sample was white (80.8%) and religious (62.2%), with an average age of 31.2 years. Just under half of the sample had some form of college degree, 37.8% had some college education without a degree, 12.5% graduated from high school or got their GED, and 3.3% did not have either a high school diploma or GED. Most of the sample was insured (87.3%), and private insurance (61.5%) was the most reported type. Over half of the sample (58.6%) belonged to households that made less than $50,000 a year, 25% made between $50,000 and $10,000, with the final 16.7% reporting an income of $100,000 or more. In terms of health status, 80% of the sample reported overall health as being good or better and 28% identified as having some form of disability. For additional descriptive data, please see Table 1. 

### 3.2. Descriptive Results

Overall, 87.4% (*n* = 24,161) of participants had visited a physician in the last year and 76.6% (*n* = 21,013) had a PCP. Of those who had a PCP, 17.8% (*n* = 3724) of participants reported that their PCP knew almost nothing about TGD health, 17.7% (*n* = 3709) that their PCP knew some things, 12.6% (*n* = 2643) that their PCP knew most things, and 15.2% (*n* = 3201) that their PCP knew almost everything. The remaining 36.7% (*n* = 7711) did not know how much their PCP knew about TGD health.

### 3.3. Regression Analyses

#### 3.3.1. Physician Visit in the Past Year 

The first column in Table 2 shows a logistic regression exploring the association between sociodemographic variables and having visited a physician in the past year. Those TGD individuals who identified as men were more likely to have gone to a physician in the past year compared to women (OR 0.74, *p* = 0.023), trans women (OR 0.62, *p* < 0.001), trans men (OR 0.72, *p* = 0.006), nonbinary/genderqueer (OR 0.42, *p* < 0.001), and cross-dressers (OR 0.24, *p* < 0.001). In terms of race and ethnicity, those who were biracial or multiracial (OR 0.73, *p* = 0.003), Latino/a or Hispanic (OR 0.84, *p* = 0.041), and identified with additional races or ethnicities (OR 0.71, *p* = 0.034) were less likely than white individuals to have visited a physician in the past year. Additionally, those who had insecure housing (OR 0.71, *p* < 0.001) were 30% less likely to visit a physician in the past year compared to those who had personal, non-congregate housing. Participants who reported being religious were more likely to have seen a physician in the past year than those who were not religious (OR 1.22, *p* < 0.001). Participants who had some college without a degree (OR 1.59, *p* = 0.003), a Bachelor’s degree, some graduate school without a degree (OR 2.48, *p* < 0.001), a Master’s degree (OR 2.25, *p* < 0.001), and a doctorate or other professional degree (OR 1.73, *p* = 0.024) were more likely to have gone to a physician in the past year compared to those without a high school diploma or GED. All forms of insurance were significantly related to having visited a physician; participants who had private (OR 3.75, *p* < 0.001), public (OR 4.89, *p* < 0.001), military (OR 3.33, *p* < 0.001), and multiple forms of insurance (OR 5.23, *p* < 0.001) all visited a physician more often than those who did not have insurance. Those who had one or more disabilities were almost two times more likely to report a physician visit in the past year compared to those without any disabilities (OR 1.83, *p* < 0.001). Older individuals were more likely than younger individuals to have gone to the physician in the past year (OR 1.01, *p* < 0.001). Lastly, those who indicated a better overall health were more likely to report a physician visit in the past year compared to those who indicated poorer health (OR 1.08, *p* = 0.004).

#### 3.3.2. Having a PCP

The second column of Table 2 illustrates a logistic regression that explores the association between having a PCP and the various sociodemographic predictor variables. Compared to those who identified as trans men, participants that identified as trans women (OR 0.81, *p* = 0.029), nonbinary/genderqueer (OR 0.72, *p* < 0.001), or cross-dressers (OR 0.40, *p* < 0.001) were less likely to have a PCP. Older participants (OR 1.03, *p* < 0.001) and participants who had served in the military (OR 1.28, *p* = 0.008), identified as religious or spiritual (OR = 1.16, *p* = 0.001), had one or more disabilities (OR 1.42, *p* < 0.001), and reported better overall health (OR 1.13, *p* < 0.001) were all more likely to have a PCP, while participants with insecure housing (OR 0.63, *p* < 0.001) were less likely. Having private (OR 5.84, *p* < 0.001), public (OR 7.34, *p* < 0.001), military (OR 7.17, *p* < 0.001), and multiple (OR 6.70, *p* < 0.001) insurances were associated with between 5.5 and 7.5 times the odds of having a PCP compared to not having insurance.

#### 3.3.3. PCP TGD Knowledge

Our analysis shown in the third column of Table 2 is an ordered logistic regression among those with a PCP, exploring the association between sociodemographic predictors and how knowledgeable a participant’s PCP was about TGD health. Identifying as non-binary/genderqueer (OR 0.47, *p* < 0.001) or as a cross-dresser (OR 0.32, *p* < 0.001) was associated with reporting lower levels of PCP TGD health knowledge, while identifying as a transgender woman (OR 1.18, *p* = 0.049) was associated with higher levels of PCP TGD health knowledge. Lesbian (OR 0.83, *p* = 0.025), bisexual (OR 0.85, *p* = 0.040), and pansexual (OR 0.82, *p* = 0.021) participants, as well as those who identified with an additional sexual orientation (OR 0.80, *p* = 0.021) had reduced odds of having a knowledgeable PCP compared to straight peers, while queer (OR 1.28, *p* = 0.002) participants had higher odds. Asian/Asian American (OR 1.25, *p* = 0.032) and Black/African American participants (OR 1.64, *p* < 0.001) were more likely than white participants to have a TGD knowledgeable PCP. Military service (OR 0.83, *p* = 0.013) was associated with reduced odds of TGD PCP knowledge, while age (OR 1.01, *p* < 0.001) and overall health (OR 1.38, *p* < 0.001) were associated with increased odds. Those in congregate housing were also more likely than those in personal, non-congregate housing to have a TGD knowledgeable PCP (OR 1.85, *p* = 0.011). Notably, the only form of insurance that was significantly different from no insurance was military insurance (OR 0.56, *p* < 0.001), which was significantly associated with reduced PCP TGD health knowledge. Additionally, neither education nor income were significant in this model.

## 4. Discussion

We can see some interesting trends across healthcare experiences with doctors and PCPs when we look at all three regressions side by side. All genders were less likely to have seen a doctor in the past year compared to men, and similarly, all genders (except trans men) were less likely to have a PCP than men. This trend changed slightly when it comes to having a trans knowledgeable PCP; trans women were more likely to have one than trans men, while nonbinary participants and cross dressers were less likely. Other genders were not significant in this analysis. This confirms previous findings that there are gender differences in healthcare experiences within the TGD population [51], and these need to be explored more fully to understand unique experiences and needs across various genders when it comes to TGD healthcare. 

When looking at sexual orientation, asexual/demisexual and pansexual individuals were less likely to have visited a doctor in the past year than straight individuals. While for having a PCP, only those with additional orientations had a significant finding, which was again less likely than straight individuals to have a PCP. Interestingly, regarding having a trans knowledgeable PCP, queer respondents were actually more likely to have one than straight respondents, while bisexual, lesbian, pansexual, and those with additional orientations were significantly less likely. There is very little research on the experience of TGD individuals who are also queer, lesbian, bisexual, and pansexual; much of the extant literature focuses on straight TGD individuals and gay TGD individuals. More research is needed to explore these within group differences, and more education is needed for providers to understand that the TGD population is not a monolithic group, and that they should be aware of not only being inclusive to gender minorities, but sexual minorities as well.

In looking at race and ethnicity, those who were biracial/multiracial, Latino/a/Hispanic, and who had additional racial/ethnic identities were less likely to have visited a doctor in the past year than their white counterparts. On the other hand, Asian/Asian American and Black/African participants were more likely than white respondents to have a trans knowledgeable PCP. Race and ethnicity were not significant for having a PCP in general. Future research is needed specifically with these sub-populations of TGD individuals to better understand their experiences, barriers, and facilitators not only to healthcare in general, but also to trans knowledgeable PCPs.

Perhaps not surprisingly, those experiencing insecure housing were less likely to have visited a doctor in the past year than those who had personal housing. Less expected was that respondents who lived in congregate housing settings were significantly more likely to have a trans knowledgeable PCP. Future research should consider exploring different types of congregant care settings to better understand these findings. Housing was not significant regarding having a PCP. Religious individuals were more likely both to have visited a doctor in the past year as well as have a PCP than those who reported not being religious, but there were no significant findings about having a trans knowledgeable PCP.

Education was significant regarding having visited a doctor in the past year, with all educational experiences (other than a high school diploma/GED and an associate’s degree) being connected to being more likely to have seen a doctor in the past year. Interestingly, there was no significance across education experiences for having a PCP or having a trans knowledgeable PCP. It was also interesting that an income of $50,000 and higher indicated a higher likelihood of having seen a doctor in the past year and having a PCP, compared to those with no income, while income was not significant for having a trans knowledgeable PCP. This is particularly of interest, as simply having some income does not change the likelihood of visiting the doctor or having a PCP, but rather, income needs to be $50,000 a year or higher to increase the likelihood. It also showcases that while income may make it easier for TGD individuals to have a PCP and go to the doctor, a lack of trans knowledgeable providers cuts across class status. 

Those with a military history were significantly more likely to have seen a doctor in the past year and have a PCP as compared to those with no military history, and were also less likely to have a trans knowledgeable PCP. Given many documented issues of challenges faced by TGD veterans in the VA system [46,47], this is in line with extant research, and indicates a need for better training for military connected healthcare providers, especially as most active service members and veterans do not have the option (whether financially, location wise, etc.) to seek more knowledgeable and/or affirming providers elsewhere. 

With every year of increase in age, there was a significant increase in likelihood across all three outcome variables, likely because as people age, they have access to more resources (both fiscal and community based), as well as may have received more messaging about the importance of regular doctor visits and having a PCP. Given that older individuals are also more likely to have a trans knowledgeable PCP, this could be an opportunity for programs that create intergenerational spaces in TGD communities, where “trans elders” (this term is often used to refer to TGD individuals aged 50+ [51]) could share their experiences in healthcare systems with younger TGD individuals, helping to connect them to knowledgeable providers. However, it is also possible that older TGD individuals may have a different definition of what it means to be trans knowledgeable than younger individuals. More research at the intersection of TGD identity and aging is needed, particularly within the health and healthcare domain.

Furthermore, not surprisingly, having better overall health was associated with having seen a doctor in the past year, having a PCP, and additionally having a trans knowledgeable PCP. This is likely a bidirectional relationship; having a regular doctor who knows about your body and its needs is more likely to lead to better overall health and having better overall health means that you have more capacity to find a PCP who is knowledgeable about your healthcare needs, and you are likely to go see them more. It is important that future research delves more deeply into this relationship, as it could be one of the keys to increasing the overall health and regular healthcare visits of the TGD population.

Having a disability resulted in being more likely to have seen a doctor in the past year as well as having a PCP, which is not a surprise given the additional health needs of disabled individuals. However, disability was not significantly related to having a trans knowledgeable PCP. Given that previous research has indicated disabled TGD individuals experience higher rates of anti-trans discrimination than their non-disabled counterparts [52], this indicates a need for more training for all healthcare providers about both trans related healthcare and being a PCP for those with disabilities, chronic illnesses, and other such medical conditions. Disabled TGD people should not have to choose between a trans knowledgeable PCP and a disability aware PCP.

In terms of having insurance, those participants with insurance were approximately three to five times more likely to have seen a doctor in the past year and approximately six to seven times more likely to have a PCP than their counterparts with no insurance. However, there was little in the way of significant findings regarding insurance and trans knowledgeable PCPs; those with military insurance were half as likely to have a trans knowledgeable PCP than those with no insurance, again affirming the need for more TGD affirming policies and continuing education training for military and VA providers. These findings also showcase that while there absolutely should be a push for TGD people to get access to being insured, as it will increase their likelihood of seeing a doctor and having a PCP, insurance alone is not enough to be able to access a knowledgeable PCP. Insurance companies should ensure that their panels of providers include trans knowledgeable PCPs in additional to gender-affirming specialists, that there are an appropriate number of these providers (rather than only one or two for an entire area), and that their plans cover all types of gender-affirming care.

Every primary care provider, regardless of location, urbanicity, size of practice, etc., should be prepared for and knowledgeable about TGD related healthcare. Medical schools need to integrate more of this into their curriculums, grand rounds need to cover these topics, and they should be a required component of ongoing medical education. Policies need to shift at the organization, local, state, and federal level to ensure that knowledge of gender-affirming care and basic knowledge on prescribing for and treating TGD patients is included not only as part of training in medical schools, but on an ongoing basis, as change often happens both regarding TGD identities and the medications used to support individuals who have opted for a medical transition of any sort. 

Moreover, it is crucial that this education be intersectional across gender, race, sexual orientation, age, disability status, housing status, income, and educational achievement. TGD individuals deserve not only access to care, which has been noted in research throughout the past decade or two, but specifically care that is knowledgeable about their identities, experiences, and needs that may be specific to their TGD status. Using an intersectional approach that understands culturally responsive care must take into account all of the identities and experiences of TGD clients, rather than asking them to choose their most salient identity when attempting to access care. Anything less than this is a failure of medicine for the TGD population and a violation of the Hippocratic oath.

### Limitations

There are, however, a few limitations to note. The study used cross-sectional survey data, precluding any claims of directionality of correlations. Findings are limited to a snapshot in time of when the survey was taken. Furthermore, as with any secondary data analysis, the researchers did not have direct input on survey questions, and it is possible that some variables could have been more appropriately measured. For example, the USTS did not include a universal question asking all participants whether they had a PCP. Rather, the survey asked participants different questions about who they sought their primary care from (i.e., routine health provider or trans-related health provider). These questions were combined into an overall measure of whether participants had a PCP, but it is possible that participants may have interpreted these individual questions differently, potentially impacting responses. Additionally, although the sample was large, it was not representative, limiting some of its generalizability. Specifically, the sample was younger, more educated, and had more white people than the US TGD population is believed to have [53]. To ameliorate this limitation, weighting was used to adjust the sample to be more in line with the larger TGD population. Using the weighted data helped to adjust for some of these issues, but this continues to be a challenge with many of the larger samples of TGD individuals. Until gender is included in a more robust way on the U.S. Census and every state’s health survey, it is impossible to assess how representative these samples are. 

## 5. Conclusions

TGD individuals often face health and healthcare challenges, whether from issues related to lack of access to basic health or discrimination in medical settings. Many may have trouble finding a trans knowledgeable provider to be their PCP. This study explored how different sociodemographic identities and their experiences may increase or decrease TGD individuals’ trips to the doctor, having a PCP, and having a trans knowledgeable PCP. Providers of all types, but especially those who offer primary care, should be educated on and prepared to support the needs of the TGD population, and we as a society need to do a better job in creating policies, offering training, and disrupting systems of oppression in order to better serve this marginalized population.

## Figures and Tables

**Table 1:** Sample demographics (total *N* = 27,715).

Demographics	% (n)		% (n)
Gender Identity:		Education	
Woman	13.51 (3743)	Less than High School/GED	3.27 (906)
Trans Woman	20.34 (5636)	High School or GED	12.51 (3467)
Man	8.26 (2288)	Some College, no Degree	37.84 (10,486)
Trans Man	21.13 (5857)	Associate’s Degree	8.42 (2333)
Non-binary/Genderqueer	34.15 (9464)	Bachelor’s Degree	19.09 (5291)
Cross-dresser	2.62 (727)	Some Grad School, No Degree	5.96 (1652)
Sexual Orientation:		Master’s Degree	9.24 (2562)
Asexual/Demisexual	11.80 (3271)	PhD or Professional Degree	3.67 (1018)
Bisexual	14.90 (4129)	Have Insurance	
Gay	4.75 (1316)	No Insurance	12.73 (3504)
Heterosexual/Straight	12.13 (3363)	Private Insurance	61.50 (16,933)
Lesbian	10.96 (3057)	Public Insurance	11.17 (3074)
Pansexual	18.24 (5056)	Military Insurance	2.18 (601)
Queer	20.59 (5706)	Multiple Insurance Types	12.42 (3420)
Additional Orientation	6.63 (1837)	Household Income	
Race/Ethnicity		No Income	3.94 (996)
American Indian	1.07 (297)	$1 to $9999	12.31 (3113)
Asian/Asian American	2.52 (699)	$10,000 to $24,999	19.87 (5027)
Biracial/Multiracial	4.65 (1290)	$25,000 to $49,999	22.47 (5683)
Black/African American	2.79 (773)	$50,000 to $99,999	24.75 (6262)
Latino/a/Hispanic	5.24 (1453)	$100,000 or more	16.66 (4215)
White/European American	80.76 (22,382)	Military	8.69 (2403)
Additional Race/Ethnicity	2.96 (821)	Overall Health	
Housing: *Ref Personal Housing*		Poor	5.07 (1404)
Personal Housing	90.55 (24,980)	Fair	15.66 (4336)
Congregate Housing	0.51 (142)	Good	33.77 (9351)
Insecure Housing	8.93 (2464)	Very Good	33.44 (9261)
Religious	62.18 (10,460)	Excellent	12.06 (3339)
Age (Mean; SD)	31.23; 13.52	Disabled	28.08 (7764)

**Table 2:** Logistic and ordered regression models of factors correlated with having seen a doctor in the past year, having a PCP, and how knowledgeable a PCP is about TNB health.

	Regression 1 Past-Year Doctor *N* = 27,715	Regression 2 Have a PCP *N* = 27,715	Regression 3 PCP TNB Knowledge *N* = 13,295
Gender Identity: (*Ref Man)*			
Woman	0.74 [0.57–0.96] *	0.82 [0.67–0.99] *	0.95 [.80–1.12)
Trans Woman	0.62 [0.49–0.79] ***	0.81 [0.67–0.98] *	1.18 [1.00–1.39) *
Trans Man	0.72 [0.57–0.91] **	0.93 [0.78–1.11]	0.93 [0.80–1.09)
Non-binary/Genderqueer	0.42 [0.34–0.52] ***	0.72 [0.61–0.86] ***	0.47 [0.39–0.57) ***
Cross-dresser	0.40 [0.17–0.33] ***	0.42 [0.30–0.53] ***	0.32 [0.22–0.47) ***
Sexual Orientation: (*Ref Heterosexual)*			
Asexual/Demisexual	0.74 (0.59–0.93) *	0.91 [0.76–1.11]	0.86 [0.71–1.04)
Bisexual	0.82 (0.66–1.01)	0.87 [0.73–1.04]	0.85 [0.72–0.99) *
Gay	0.85 (0.64–1.13)	0.99 [0.78–1.25]	1.12 [0.92–1.37)
Lesbian	0.82 (0.65–1.04)	0.83 [0.69–1.01]	0.83 [0.70–0.98) *
Pansexual	0.78 (0.63–0.96) *	0.86 [0.73–1.01]	0.82 [0.69–0.97) *
Queer	1.11 (0.90–1.37)	0.91 [0.77–1.07]	1.28 [1.10–1.50) **
Additional Orientation	0.84 (0.65–1.09)	0.80 [0.65–0.9]*	0.80 [0.65–0.96) *
Race/Ethnicity *(Ref White)*			
American Indian	1.35 (0.87–2.00)	1.20 [0.87–1.66]	0.99 [0.73–1.34]
Asian/Asian American	0.93 (0.71–1.21)	1.11 [0.90–1.38]	1.25 [1.02–1.53] *
Biracial/Multiracial	0.73 (0.59–0.90) **	0.91 [0.77–1.08]	1.17 [0.97–1.40]
Black/African American	0.87 (0.70–1.09)	1.00 [0.83–1.22]	1.64 [1.32–2.03] ***
Latino/a/Hispanic	0.84 (0.71–0.99) *	1.08 [0.94–1.25]	1.13 [0.96–1.33]
Additional Race/Ethnicity	0.71 (0.52–0.97) *	1.04 [0.82–1.32]	1.25 [0.99–1.56]
Housing: (*Ref Personal Housing)*			
Congregate Housing	1.36 (0.55–3.37)	1.11 [0.59–2.10]	1.84 [1.15–2.97] *
Insecure Housing	0.71 (0.60–0.84) ***	0.63 [0.55–0.72] ***	0.96 [0.80–1.15]
Religious	1.22 (1.09–1.36) ***	1.16 [1.07–1.27] **	1.03 [0.94–1.13]
Education: (*Ref Less than Diploma or GED*)			
HS Diploma or GED	1.07 (0.78–1.48)	0.82 [0.61–1.09]	0.99 [0.64–1.52]*
Some College, No Deg	1.59 [1.17–2.15] **	1.03 [0.78–1.36]	0.80 [0.53–1.21]
Associate’s Degree	1.28 [0.90–1.81]	0.91 [0.67–1.24]	0.92 [0.60–1.40]
Bachelor’s Degree	1.58 [1.15–2.18] **	0.86 [0.65–1.15]	1.14 [0.75–1.72]
Some Grad School, No Deg	2.25 [1.67–3.67] ***	1.13 [0.82–1.55]	1.06 [0.69–1.62]
Master’s Degree	2.25 [1.55–3.27] ***	1.07 [0.78–1.47]	0.99 [0.65–1.51]
PhD or Professional Deg	1.73 [1.07–2.79] *	0.97 [0.66–1.44]	0.82 [0.53–1.28]
Insurance: (*Ref No Insurance*)			
Private Insurance	3.75 [3.29–4.27] ***	5.84 [5.20–6.56] ***	0.99 [0.83–1.18]
Public Insurance	4.89 [3.90–6.12] ***	7.34 [6.18–8.73] ***	1.10 [0.89–1.37]
Military Insurance	3.33 [2.17–5.10] ***	7.17 [5.00–10.27] ***	0.55 [0.41–0.76] ***
Multiple Insurance Types	5.23 [4.24–6.45] ***	6.70 [5.70–1.87] ***	1.04 [0.85–1.26]
Household Income: (*Ref No Income*)			
$1 to $9999	1.07 [0.80–1.43]	0.86 [0.68–1.10]	1.01 [0.70–1.45]
$10,000 to $24,999	1.06 [0.81–1.40]	0.90 [0.72–1.13]	1.16 [0.83–1.63]
$25,000 to $49,999	1.22 [0.92–1.61]	1.10 [0.87–1.38]	1.20 [0.86–1.68]
$50,000 to $99,999	1.54 [1.16–2.05] **	1.49 [1.18–1.89] **	1.18 [0.84–1.64]
$100,000 or more	1.29 [1.40–2.59] ***	1.92 [1.50–2.46] ***	1.14 [0.81–1.60]
Military	1.24 [0.98–1.56]	1.28 [1.07–1.54] **	0.83 [0.72–0.96] *
Age	1.01 [1.00–1.02] ***	1.03 [1.03–1.04] ***	1.01 [1.01–1.01] ***
Overall Health	1.08 [1.03–1.14] **	1.13 [1.08–1.18] ***	1.38 [1.32–1.45] ***
Disabled	1.83 [1.60–2.08] ***	1.43 [1.28–1.59] ***	1.02 [0.92–1.14]

NOTE: *** ≥ 0.001. ** ≥ 0.01. * ≥ 0.05.

## Data Availability

The data that support the findings of this study are available on request from the corresponding author or through the United States Transgender Study research team.
